# The new invasive mosquito species *Aedes koreicus* as vector-borne diseases in the European area, a focus on Italian region: What we know from the scientific literature

**DOI:** 10.3389/fmicb.2022.931994

**Published:** 2022-07-25

**Authors:** Sonia Ganassi, Antonio De Cristofaro, Dalila Di Criscio, Sonia Petrarca, Chiara Leopardi, Antonio Guarnieri, Laura Pietrangelo, Noemi Venditti, Roberto Di Marco, Giulio Petronio Petronio

**Affiliations:** ^1^Department of Agricultural, Environmental and Food Sciences (DiAAA), Università degli Studi del Molise, Campobasso, Italy; ^2^Department of Medicine and Health Science (DiMeS), Università degli Studi del Molise, Campobasso, Italy

**Keywords:** Korean bush mosquito, nematodes, *Flavivirus*, *Alphavirus*, hematophagous arthropod

## Abstract

The increased mobility of goods, people, and animals worldwide has caused the spread of several arthropod vectors, leading to an increased risk of animal and human infections. *Aedes koreicus* is a common species in South Korea, China, Japan, and Russia. Due to its cold-resistant dormant eggs, the adults last from the late summer until the autumn seasons. For these reasons, it seems to be better adapted to colder temperatures, favoring its colonization of hilly and pre-alpine areas. Its first appearance in Europe was in 2008 in Belgium, where it is currently established. The species was subsequently detected in Italy in 2011, European Russia, Germany, the Swiss–Italian border region, Hungary, Slovenia, Crimea, Austria, the Republic of Kazakhstan, and the Netherlands. The role of *A. koreicus* in the transmission of vector-borne pathogens remains unclear. The available scientific evidence is very old, often not available in English or not indexed in international databases, and therefore difficult to find. According to the literature reviewed, *A. koreicus* can be considered a new invasive mosquito species in Europe, establishing populations on the European continent. In addition, experimental evidence demonstrated its vector competence for both *Dirofilaria immitis* and *Chikungunya* and is relatively low for ZIKA but not for *Western Nile Virus*. On the other hand, even if the field evidence does not confirm the experimental findings, it is currently not possible to exclude with absolute certainty the potential involvement of this species in the spread, emergence, or re-emergence of these vector-borne disease agents.

## Introduction

**Figure 1:**
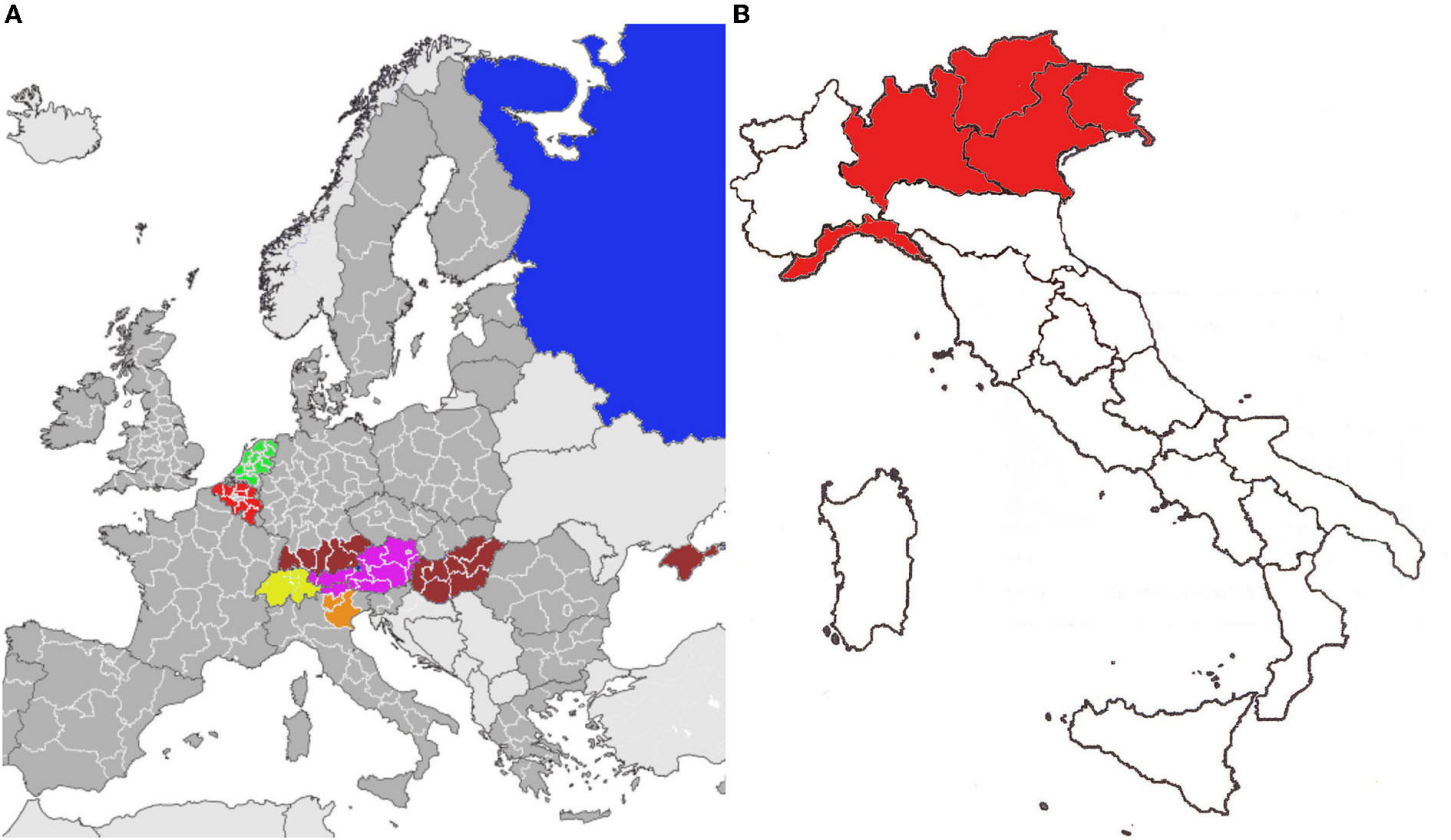
*Aedes koreicus* evidence. **(A)** The first evidence in the European area (colored area represents *A. koreicus* first detection along with the first recording date). Red 2008: Belgium (Versteirt et al., 2012); orange 2011: Italy (Valbelluna, Province of Belluno, Veneto Region) (Capelli et al., 2011); yellow 2013: Switzerland (Suter et al., 2015); dark blue 2014: European Russia (Bezzhonova et al., 2014); brown 2016: Hungary (Kurucz et al., 2016), Germany (Southern Germany) (Werner et al., 2016), and Crimean Peninsula (Ganushkina et al., 2020); violet 2018: Austria (Fuehrer et al., 2020); and green 2021: the Netherlands (Teekema et al., 2022). **(B)** Northern Italy diffusion from 2011 to 2021. Red: Veneto, Trentino Alto Adige, Friuli Venezia Giulia, Lombardia, and Liguria (Montarsi et al., 2013, 2015b; Marcantonio et al., 2016; Baldacchino et al., 2017b; Gradoni et al., 2021; Negri et al., 2021).

*Aedes koreicus* (Edwards, 1917), also known as the Korean bush mosquito, is naturally distributed in South Korea, China, Japan, and Russia (Knight, 1968; Gutsevich et al., 1974; Tanaka et al., 1979). The first occurrence in Europe was recorded in Belgium in 2008, where it is currently established (Versteirt et al., 2012, 2014). The species was subsequently detected in Italy in 2011 (Capelli et al., 2011; Marcantonio et al., 2016; Ballardini et al., 2019; Negri et al., 2021), European Russia (Bezzhonova et al., 2014), Germany (Werner et al., 2016; Pfitzner et al., 2018; Zotzmann et al., 2019; Hohmeister et al., 2021), Swiss–Italian border region (Suter et al., 2015), Hungary (Kurucz et al., 2016), Slovenia (Kalan et al., 2017), Sochi, Russia (Ganushkina et al., 2016), Crimea (Kovalenko and Tikhonov, 2019; Ganushkina et al., 2020), Austria (Fuehrer et al., 2020), and Republic of Kazakhstan (Andreeva et al., 2021) (see Figure 1) for further details). *A. koreicus* is nowadays established (Jansen et al., 2021) in all the countries mentioned above except Slovenia and Switzerland (ECDC, 2020). In 2021, the first finding of *A. koreicus* larvae in the Netherlands was made (Teekema et al., 2022). Until now, nothing is known about the initial pathways of the introduction of *A. koreicus* into the different European areas, although international trade has been suggested as a possible route (Versteirt et al., 2012). For what concerned *A. koreicus* arrival time in Italy, the second European *A. koreicus* population was detected in 2011 in Belluno province. However, it has been supposed that this species has been present for several years (Pfitzner et al., 2018) (Figure 1).

The females lay their drought-resistant eggs in artificial breeding sites such as containers, like *Aedes* (*Stegomyia*) *albopictus* (Skuse) and *Aedes* (*Hulecoeteomyia*) *japonicus japonicus* (Theobald), so its introduction could be due to the trade of small containers, ornamental plants or used tire (Versteirt et al., 2012; Medlock et al., 2015; Kampen et al., 2017; Ibáñez-Justicia et al., 2020). *A. koreicus* and the Japanese bush mosquito *A. japonicus* lived in sympathy and, for a long time, were confused. Indeed, according to phylogenetic studies, both mosquito species belong to the same monophyletic group (Miyagi, 1971; Cameron et al., 2010; Hohmeister et al., 2021). However, *A. koreicus* adults differ from the latter in having a pale basal band at the hind tarsomeres IV, the typical scutal pattern, the presence of a subspiracular patch of pale scales, and the base of the posterior femur is completely pale (Cameron et al., 2010; Werner et al., 2016; Pfitzner et al., 2018).

*Aedes koreicus* has settled in areas partially occupied by other mosquito species, including its most likely competitor, *A. albopictus*, which uses similar larval habitats for its development (Montarsi et al., 2013). Although larval coexistence between these two species is possible, it was not expected. Indeed, field observations carried out in the Province of Belluno, and the neighboring provinces demonstrated that *A. albopictus* larvae number was higher than the *A. koreicus* ones during the fall and toward the south. Moreover, they were often found alone and developed earlier than *A. albopictus* ones. According to the authors, northern Italy is highly likely to be invaded by *A. koreicus* in the next decade and other parts of Europe (Montarsi et al., 2013, 2015b). Laboratory experiments have also revealed a weak larval competition between *A. albopictus* and *A. koreicus*, with a slight advantage in favor of the first one (Baldacchino et al., 2017a).

At the end of the annual season, when daylight becomes shorter, *A. koreicus* females, like other females of the *Aedes* species, lay desiccated and cold-resistant eggs that can survive during the winter and hatch in the spring (Medlock et al., 2015). Compared to *A. albopictus, A. koreicus* dormant eggs are more resistant to cold temperatures. Furthermore, adults have a more remarkable persistence during the late summer and autumn seasons, and the species is active earlier. For these reasons, *A. koreicus* seems to be better adapted to colder temperatures, favoring its colonization of hilly and prealpine areas (Knight, 1947; Miyagi, 1971; Capelli et al., 2011; Montarsi et al., 2013; Baldacchino et al., 2017a). In particular, laboratory trials demonstrated that *A. koreicus's* optimal thermal range is placed between 23°C and 28°C, and the authors found that warmer seasonal temperatures provoke an increase in values of the adult abundance curve (Marini et al., 2019). This last evidence is especially relevant concerning climate change causing increased temperatures in the Alpine area (Coppola et al., 2018).

Moreover, *A. koreicus's* ability to colonize areas with harsh winter temperatures allows the species to avoid competition with other species, such as *A. albopictus*, showing a considerable advantage in terms of number and speed of replication (Baldacchino et al., 2017a).

Several studies indicate that *A. koreicus* is well adapted to urban settlements and colonize gardens and urban areas, where various artificial containers can be found as breeding sites (Gutsevich et al., 1974; Tanaka et al., 1979; Montarsi et al., 2013). However, this species was also frequently found in natural habitats, like forests, far from human settling (Baldacchino et al., 2017b; Pfitzner et al., 2018), suggesting that *A. koreicus* can complete its life cycle by feeding on animals other than humans (Montarsi et al., 2013). Until now, only three species of mammals (*Homo sapiens, Canis lupus*, and *Bos taurus*) have been detected as hosts, and adult females seem to bite humans mainly during the daytime (Montarsi et al., 2014, 2015b; Tripepi, 2014; Cebrián-Camisón et al., 2020).

According to the European Center for Disease Prevention and Control (ECDC, 2020), *A. koreicus* in transmitting vector-borne pathogens remains unclear. Thus, this narrative review aims to provide a broad view in light of the latest scientific evidence about *A. koreicus* vector competence, focusing on the re-emergence of vector-borne diseases in the European area. The bibliographic research for scientific papers specialized in the field of interest was conducted from 1st January 2000 to 31st January 2022 on PubMed (the MEDLINE database), using the following keyword: *Aedes koreicus*. As a preliminary result, more than 57 documents were found. Of these, 8 papers, 5 on filed evidence and 3 on laboratory experiments, were selected for review due to their relevance. The search and selection criteria for reviewed scientific articles can be found in Supplementary Figure S1.

## Literature review

The selected manuscripts were subdivided according to the study type (namely, field or experimental) and reviewed according to the publication date (from oldest to most recent).

### Field evidence

Concerning field research data, the results are not entirely consistent. Of the five publications reviewed, only one by Kurucz et al. (2018) detected the presence of filaria DNA from captured mosquitoes. A possible explanation of these results can be found in how these studies were carried out and their procedural limitations.

According to Lee et al. (2007), the low number of mosquitoes collected from Gyeonggi Province and Gangwon Province in the Republic of Korea can justify the low number of *A. koreicus* that was retrieved in the study. Among the limiting factors found by the authors were the trapping methods and the sampling periodicity (only one season, from 25th May to 25th September, was considered). For these reasons, it was impossible to determine the species' vector competence and the prevalence of infection in the mosquito population. Despite the negative results (Supplementary Table 1), the involvement of *A. koreicus* as a vector of *Dirofilaria immitis* and *Dirofilaria repens* could not be excluded (Lee et al., 2007).

Another single season (May–October 2009) surveillance study, conducted by Cho et al. (2012) in the Republic of Korea over an extensive area previously considered endemic for filariasis, did not detect *Brugia malayi* DNA in a large sample of captured mosquitoes (Supplementary Table 1). Even if these data demonstrated the disease eradication from previously endemic areas, given the many vector species isolated, the authors pointed out the potential risk of re-emerging filariasis. In addition, the small number of *A. koreicus* captured (Supplementary Table 1) did not allow valid conclusions about the vector status of this species (Cho et al., 2012).

On the other hand, between 2016 and 2017, Kurucz et al. collected 68 specimens of *A. koreicus* adult female mosquitoes from 46 different pools out of 1,123 near the city of Pécs (Baranya County, Hungary). Molecular investigation for filarial DNA revealed 25 specimens positive for nematodes. As this is the first recent field study to detect the *D. immitis* DNA, further research confirming the possible vector role of this mosquito is needed. However, the presence of this species near urban areas could facilitate the spread of these vector-borne pathogens (Kurucz et al., 2018).

The first Italian record of *A. koreicus* was reported in Genoa in September 2015, when a male specimen was caught, and genetic analysis confirmed the identity of the species. Mosquito sampling, conducted in different periods until 2018, led to trapping some mosquito species, and morphological and molecular studies allowed us to identify some samples as *A. koreicus* females.

A subsequent biomolecular assay to detect *Flavivirus* infection showed the DNA absence in females. The authors explained this result by the low density of the species in the sampling area and the low meeting rate with infected hosts (Ballardini et al., 2019).

Similar evidence was reported from a 3-year surveillance study conducted by Jegal et al. (2020) in several provinces of the Korean republic (Jegal et al., 2020). Even though there was geographical proximity to the previous studies reviewed (Lee et al., 2007; Cho et al., 2012), the research procedures were implemented to obtain more comprehensive epidemiological results (i.e., mosquito collection procedures and *Flavivirus* detection analysis). Thus, sampling was carried out for three consecutive seasons (24 h every fortnight from March to November 2016–2018), and the trap type was adjusted according to the locations analyzed (downtown or cowshed). Furthermore, the authors established the distribution of total population densities of each captured mosquito species according to the collection time. This could explain the significant increase in *A. koreicus* specimens caught in the field (Supplementary Table 1). Molecular analyses on mosquitoes showed the absence of DNA belonging to the three *Flavivirus* species investigated (WNV, JEV, and dengue fever virus). Nevertheless, even in this study, the possible role of *Ae koreicus* as a vector was not wholly rejected (Jegal et al., 2020).

### Experimental evidence

In a detailed laboratory study conducted by Montarsi et al. (2015), *A. koreicus* adults were experimentally administered with *D. immitis* microfilariae. Parasites at the third larval stage (L3) have been detected in the mosquito's malpighian tubules, thorax, salivary glands, palp, and proboscis, demonstrating that infective larvae could develop within the species. The authors also observed L3 emerging from the mosquito proboscis. However, the L3 host transmission would confirm the role of *A. koreicus* as a *D. immitis* vector and requires further experimental evidence (Montarsi et al., 2015a).

In laboratory experiments, Ciocchetta et al. (2018) explored the potential of *A. koreicus* to transmit CHIKV “*La Reunion”* under different temperature regimes: at a constant temperature of 23°C and fluctuating temperatures (12–27°C). These fluctuations simulated a typical summer in Belluno, Italy, where thriving populations of *A. koreicus* are established. The experiments highlighted that, at fluctuating temperatures, despite the very favorable infection conditions, CHIKV “*La Reunion”* was detected in a tiny percentage of mosquito bodies; the virus dissemination to the legs and wings was also recorded on a low number of mosquitoes, and for the salivary dissemination. However, CHIKV “*La Reunion”* disseminates to the *A. koreicus* at a constant temperature from wings and legs reaching the saliva. Based on the results, *A. koreicus* may transmit CHIKV. However, only a tiny proportion of mosquitoes may vector the virus under optimal rearing temperatures, and more natural temperature fluctuations might further mitigate transmission risks (Ciocchetta et al., 2018).

Jansen et al. (2021), demonstrated that experimentally infected *A. koreicus* specimens were able to transmit CHIKV, and, according to Ciocchetta et al., the transmission was temperature-dependent. The mosquitoes were capable of transmitting the virus at a higher temperature (27°C ± 5°C), with no transmission at 24°C ± 5°C. Moreover, the infection rate at the higher temperature (68.2%) was four times higher than at the lower one (17.6%). Regarding the ZIKA virus, the vector competence was relatively low (4.7%) and temperature-dependent; in particular, ZIKV transmission occurred only at a higher temperature. No transmission of WNV could be detected at both temperatures. Although able to cross the midgut barrier and infect the whole mosquito body, the authors hypothesized that the virus could not pass the salivary gland barrier. They also hypothesized that both the salivary glands' infection and the virus's escape from the tissue into the saliva failed (Jansen et al., 2021).

## Discussion

The increased mobility of goods, people, and animals worldwide has caused the spread of several arthropod vectors, leading to an increased risk of animal and human infection (Genchi et al., 2009).

In recent years, several vector-borne diseases have re-emerged and spread in Europe due to global and/or local changes that have led to the invasion of new arthropod vectors (Hendrickx and Nicolaij, 2004). The direct consequences of climatic variations include habitat alterations and a lengthened mosquito season with an increased incidence of mosquito-borne diseases (Harrus and Baneth, 2005). Mosquito survival is strongly influenced by the environmental temperature and the pathogens carried by them (Purse et al., 2005). In this scenario, *A. koreicus* adaptation to colder temperatures ensures longer persistence during late summer and autumn, leading to the colonization of hilly and prealpine areas (Knight, 1947; Miyagi, 1971; Capelli et al., 2011; Montarsi et al., 2013; Baldacchino et al., 2017a). In addition, anthropogenic factors such as water shortages due to human consumption or irrigation, pollution, insecticides, and drug-resistance development can impact the vector-borne parasites (Harrus and Baneth, 2005).

The scientific papers reviewed suggest a possible involvement of *A. koreicus* as a vector of both lymphatic filariae and *Dirofilaria* and several viruses belonging to the genus *Flavivirus* and *Alphavirus*.

In 1927, Yamada found that this species can collect microfilariae but does not allow the development of *Wuchereria bancrofti* (Yamada, 1928).

Lymphatic filariasis is an underestimated tropical disease caused by parasitic nematodes known as filarial worms. Currently, approximately 856 million people in 52 countries live in endemic areas. *B. malayi*, one of the nematode species for lymphatic filariasis, is an endemic nematode in Southeast Asia and Indonesia. It develops through four larval stages into an adult male or female, entirely within one of two host species: a mosquito vector and humans (Ghedin et al., 2004). For this reason, the possible involvement of a new mosquito species as a vector is crucial to set up the necessary preventive measures to contain infections.

Dirofilariasis is a zoonotic disease transmitted by mosquitoes (family *Culicidae* belonging to the genus *Aedes*, with domestic dogs and some wild canids as definitive hosts. In 1938, Feng et al. experimentally proved the transmission of *D. immitis* to dogs (Feng, 1938). The *Dirofilaria* species reviewed in this article (*D. immitis* and *D. repens* are occasionally transmitted to humans through the bites of infected mosquitoes. Although *D. repens* is the principal agent of human dirofilariasis, causing a subcutaneous infection, *D. immitis* larvae can occasionally encapsulate in lung tissue or, more rarely, in the eyes, brain, and/or testes, producing nodules (Simón et al., 2012).

Italy is one of the European countries endemic to canine dirofilariasis, with the highest number of human cases described so far. In recent years, an underestimated increase in the number of dirofilariasis cases (*D. immitis* and *D. repens*) has been recorded (Mendoza-Roldan et al., 2021).

According to a study by S. Pampiglione et al., between 1990 and 1999, out of 60 cases of human dirofilariasis caused by *D. repens*, forty-six were from Piemonte (one of the most affected geographical areas in the world), while the remaining were from Emilia-Romagna, Sardinia, Sicily, Tuscany, Apulia, and Lombardy (Pampiglione et al., 2001). In 2018–2019, eight cases were reported from Central Italy, suggesting the current increase in the spread of the parasite in the peninsula (Mendoza-Roldan et al., 2021).

Concerning *A. koreicus* virus transmission, field evidence collected in 1964 and 1966 by Russian researchers has raised the suspicion that *A. koreicus* could be a vector for the Japanese encephalitis virus (Miles, 1964; Shestakov and Mikheeva, 1966). Furthermore, experimental virus transmission was reported in Russia in 1970 (Gutsevich et al., 1974).

Although *A. koreicus* vector competence for ZIKV and WNV has been investigated by experimental studies (Ciocchetta et al., 2018; Jansen et al., 2021), only ZIKV transmission was demonstrated in laboratory conditions without any field evidence (Jansen et al., 2021). These experimental findings on vector competence agree with those obtained for the related species *A. japonicus* which can transmit ZIKV temperature-dependent (Jansen et al., 2018; Abbo et al., 2020; Glavinic et al., 2020).

According to a 2020 study by Durand et al., the first European ZIKV cases were reported in August 2019 in France (city of Hyeres). Although the infection origin was not clarified, the authors established the source of the viral strain, namely, South-East Asia (Durand et al., 2020). Since 1st February 2016, the WHO classified Italy as at moderate risk of ZIKV transmission. ZIKV is not endemic to date as no locally contracted infection has been recorded but only imported cases have been documented (WHO, 2022).

Regarding the *Alphavirus* genus, the first autochthonous outbreak of CHIKV in Europe was reported in Emilia-Romagna (Italy) in 2007 (Marano et al., 2017). In addition, a large number of confirmed autochthonous cases were reported in France from 2010 to 2014 (Zeller et al., 2016).

In Italy, in September 2017, a new outbreak of CHIKV was detected in the Lazio region (central Italy) and subsequently in Calabria (southern Italy). The activity of vector mosquitoes is mainly linked to the summer season (usually between June and October), and outbreaks could be triggered by the arrival of imported cases from endemic areas causing, consequently, secondary cases (McCormack et al., 1995; Hassoun et al., 1999; Silva and Dermody, 2017).

Along with its role as an enzootic vector, another critical aspect of mosquito-related diseases is its allergic potential. There are limited epidemiological data on the prevalence of mosquito allergy, although reactions to mosquito bites are common. Recently, studies have proven an association between unusual, largely local, or exaggerated reactions after mosquito bites and allergic diseases in children. The severity of reactions increases with age, particularly in children with an atopic background (Yavuz et al., 2021). Moreover, a family history of allergy, and atopic dermatitis (Magnifico et al., 2020), together with a self-reported allergy to mosquito bites and residence in an area of high mosquito exposure, were associated with positive IgE levels. In contrast, positive IgG levels were significantly associated with male sex, blood donation after the “mosquito season,” and residence in the area of high exposure but inversely related to self-reported asthma (Peng et al., 2002).

In conclusion, according to the reviewed literature data, *A. koreicus* can be granted as a new invasive mosquito species in Europe. Furthermore, the experimental evidence has demonstrated its vector competence for both *D. immitis*, CHIKV, and ZIKA but not for WNV (Montarsi et al., 2015a; Jansen et al., 2021). On the other hand, although field evidence did not confirm the experimental evidence, it is currently not possible to exclude with absolute certainty the potential involvement of this species in the spread and re-emergence of these vector-borne pathogens. In this scenario, it is imperative to increase research activity to conclusively understand the infectious potential of this mosquito, which is a public health threat worldwide.

## Author contributions

AD, RD, SG, and GPP contributed conception and design of the study. CL, AG, LP, and NV organized the database. SG and GPP wrote the first draft of the manuscript. DD, SP, and CL wrote sections of the manuscript. All authors contributed to manuscript revision, and read and approved the submitted version.

## Conflict of interest

The authors declare that the research was conducted in the absence of any commercial or financial relationships that could be construed as a potential conflict of interest.

## Publisher's note

All claims expressed in this article are solely those of the authors and do not necessarily represent those of their affiliated organizations, or those of the publisher, the editors and the reviewers. Any product that may be evaluated in this article, or claim that may be made by its manufacturer, is not guaranteed or endorsed by the publisher.

## References

[B1] Abbo S. R. Visser T. M. Wang H. Göertz G. P. Fros J. J. Abma-Henkens M. H.. (2020). The invasive Asian bush mosquito *Aedes japonicus* found in the Netherlands can experimentally transmit Zika virus and Usutu virus. PLoS Negl. Trop. Dis. 14, e0008217. 10.1371/journal.pntd.0008217 32282830 PMC7153878

[B2] Andreeva Y. V. Khrabrova N. V. Alekseeva S. S. Abylkassymova G. M. Simakova A. V. Sibataev A. K. (2021). First record of the invasive mosquito species *Aedes koreicus* (Diptera, Culicidae) in the Republic of Kazakhstan. Parasite 28, 52. 10.1051/parasite/2021050 34142954 PMC8212810

[B3] Baldacchino F. Arnoldi D. Lapère C. Ros,á R. Montarsi F. Capelli G.. (2017a). Weak larval competition between two invasive mosquitoes *Aedes koreicus* and *Aedes albopictus* (Diptera: Culicidae). J. Med. Entomol. 54, 1266–1272. 10.1093/jme/tjx093 28460074

[B4] Baldacchino F. Montarsi F. Arnoldi D. Barategui C. Ferro Milone N. Da Rold G.. (2017b). A 2-yr mosquito survey focusing on *Aedes koreicus* (Diptera: Culicidae) in northern Italy and implications for adult trapping. J. Med. Entomol. 54, 622–630. 10.1093/jme/tjw216 28399310

[B5] Ballardini M. Ferretti S. Chiaranz G. Pautasso A. Riina M. V. Triglia G.. (2019). First report of the invasive mosquito *Aedes koreicus* (Diptera: Culicidae) and of its establishment in Liguria, northwest Italy. Parasit Vectors 12, 1–13. 10.1186/s13071-019-3589-2 31277680 PMC6610922

[B6] Bezzhonova O. Patraman I. Ganushkina L. Vyshemirskii O. Sergiev V. (2014). The first finding of invasive species *Aedes* (Finlaya) *koreicus* (Edwards, 1917) in European Russia. *Meditsinskaia parazitologiia i parazitarnye bolezni*, 1, 16–19. 24738221

[B7] Cameron E. C. Wilkerson R. C. Mogi M. Miyagi I. Toma T. Kim H. C.. (2010). Molecular phylogenetics of Aedes japonicus, a disease vector that recently invaded Western Europe, North America, and the Hawaiian islands. J. Med. Entomol. 47, 527–535. 10.1093/jmedent/47.4.527 20695267 PMC7027316

[B8] Capelli G. Drago A. Martini S. Montarsi F. Soppelsa M. Delai N.. (2011). First report in Italy of the exotic mosquito species *Aedes* (Finlaya) *koreicus*, a potential vector of arboviruses and filariae. Parasit. Vectors 4, 1–5. 10.1186/1756-3305-4-188 21951867 PMC3203849

[B9] Cebrián-Camisón S. Martínez-de la Puente J. Figuerola J. (2020). A literature review of host feeding patterns of invasive *Aedes* mosquitoes in Europe. Insects 11, 848. 10.3390/insects11120848 33260438 PMC7760726

[B10] Cho S. H. Koo B. R. Shin H. E. Lee W. K. Jeong B. S. Chu C.. (2012). Surveillance and vector control of lymphatic filariasis in the Republic of Korea. Osong Public Health Res. Perspect. 3, 145–150. 10.1016/j.phrp.2012.07.008 24159506 PMC3738707

[B11] Ciocchetta S. Prow N. A. Darbro J. M. Frentiu F. D. Savino S. Montarsi F.. (2018). The new European invader *Aedes* (Finlaya) *koreicus*: a potential vector of chikungunya virus. Pathog. Glob. Health 112, 107–114. 10.1080/20477724.2018.1464780 29737236 PMC6056824

[B12] Coppola E. Raffaele F. Giorgi F. (2018). Impact of climate change on snow melt driven runoff timing over the Alpine region. Clim. Dyn. 51, 1259–1273. 10.1007/s00382-016-3331-0

[B13] Durand G. A. Piorkowski G. Thirion L. Ninove L. Giron S. Zandotti C.. (2020). Vector-borne transmission of the Zika virus Asian genotype in Europe. Viruses 12, 296. 10.3390/v12030296 32182748 PMC7150815

[B14] ECDC (2020). Aedes Koreicus - Factsheet for Experts. Available online at: https://www.ecdc.europa.eu/en/disease-vectors/facts/mosquito-factsheets/aedes-koreicus (accessed 2 February 2022).

[B15] Feng L. (1938). The tree hole species of mosquitoes of Peiping, China. Chin Med J 2, 503–525.

[B16] Fuehrer H.-P. Schoener E. Weiler S. Barogh B. S. Zittra C. Walder G. (2020). Monitoring of alien mosquitoes in Western Austria (Tyrol, Austria, 2018). PLoS Negl. Trop. Dis. 14, e0008433. 10.1371/journal.pntd.0008433 PMC7337398 32574163

[B17] Ganushkina L. Lukashev A. Patraman I. Razumeyko V. Shaikevich E. (2020). Detection of the Invasive Mosquito Species *Aedes* (Stegomyia) *aegypti* and *Aedes* (Hulecoeteomyia) *koreicus* on the Southern Coast of the Crimean Peninsula. J. Arthropod Borne Dis. 14, 270. 10.18502/jad.v14i3.4560 33644240 PMC7903358

[B18] Ganushkina L. A. Patraman I. V. Rezza G. Migliorini L. Litvinov S. K. Sergiev V. P. (2016). Detection of *Aedes aegypti, Aedes albopictus*, and *Aedes koreicus* in the Area of Sochi, Russia. Vector Borne Zoo. Dis. 16, 58–60. 10.1089/vbz.2014.1761 26741323

[B19] Genchi C. Rinaldi L. Mortarino M. Genchi M. Cringoli G. (2009). Climate and Dirofilaria infection in Europe. Vet. Parasitol. 163, 286–292. 10.1016/j.vetpar.2009.03.026 19398159

[B20] Ghedin E. Wang S. Foster J. M. Slatko B. E. (2004). First sequenced genome of a parasitic nematode. Trends Parasitol. 20, 151–153. 10.1016/j.pt.2004.01.011 15099548

[B21] Glavinic U. Varga J. Paslaru A. I. Hauri J. Torgerson P. Schaffner F.. (2020). Assessing the role of two populations of *Aedes japonicus japonicus* for Zika virus transmission under a constant and a fluctuating temperature regime. Parasit. Vectors 13, 1–11. 10.1186/s13071-020-04361-2 32948231 PMC7501641

[B22] Gradoni F. Bertola M. Carlin S. Accordi S. Toniolo F. Visentin P.. (2021). Geographical data on the occurrence and spreading of invasive Aedes mosquito species in Northeast Italy. Data Brief 36, 107047. 10.1016/j.dib.2021.107047 33997197 PMC8099600

[B23] Gutsevich A. Monchadskii A. Shtakel'berg A. (1974). Fauna of the USSR Diptera, Vol. 3, No. 4, Mosquitoes, family Culicidae. Jerusalem: Keter Publishing House Jerusalem Ltd. p. 408.

[B24] Harrus S. Baneth G. (2005). Drivers for the emergence and re-emergence of vector-borne protozoal and bacterial diseases. Int. J. Parasitol. 35, 1309–1318. 10.1016/j.ijpara.2005.06.005 16126213

[B25] Hassoun S. Drouet M. Sabbah A. (1999). Anaphylaxis caused by a mosquito: 2 case reports. Allerg. Immunol. 31, 285–287. 10572584

[B26] Hendrickx L. Nicolaij S. (2004). Temporal discounting and environmental risks: The role of ethical and loss-related concerns. J. Environ. Psychol. 24, 409–422. 10.1016/j.jenvp.2004.12.001

[B27] Hohmeister N. Werner D. Kampen H. (2021). The invasive Korean bush mosquito *Aedes koreicus* (Diptera: Culicidae) in Germany as of 2020. Parasit. Vectors 14, 1–12. 10.1186/s13071-021-05077-7 34772448 PMC8588644

[B28] Ibáñez-Justicia A. Smitz N. Den Hartog W. van de Vossenberg B. De Wolf K. Deblauwe I.. (2020). Detection of exotic mosquito species (Diptera: Culicidae) at international airports in Europe. Int. J. Environ. Res. Public Health 17, 3450. 10.3390/ijerph17103450 32429218 PMC7277938

[B29] Jansen S. Cadar D. Lühken R. Pfitzner W. P. Jöst H. Oerther S.. (2021). Vector Competence of the Invasive Mosquito Species *Aedes koreicus* for Arboviruses and Interference with a Novel Insect Specific Virus. Viruses 13, 2507. 10.3390/v13122507 34960776 PMC8704790

[B30] Jansen S. Heitmann A. Lühken R. Jöst H. Helms M. Vapalahti O.. (2018). Experimental transmission of Zika virus by *Aedes japonicus japonicus* from southwestern Germany. Emerg. Microb. Infect. 7, 1–6. 10.1038/s41426-018-0195-x 30482893 PMC6258727

[B31] Jegal S. Jun H. Kim-Jeon M. D. Park S. H. Ahn S. K. Lee J.. (2020). Three-year surveillance of culicine mosquitoes (Diptera: Culicidae) for flavivirus infections in Incheon Metropolitan City and Hwaseong-si of Gyeonggi-do Province, Republic of Korea. Acta Trop. 202, 105258. 10.1016/j.actatropica.2019.105258 31733189

[B32] Kalan K. Šušnjar J. Ivovi ć V. Buzan E. (2017). First record of *Aedes koreicus* (Diptera, Culicidae) in Slovenia. Parasitol. Res. 116, 2355–2358. 10.1007/s00436-017-5532-9 28624875

[B33] Kampen H. Schuhbauer A. Walther D. (2017). Emerging mosquito species in Germany—a synopsis after 6 years of mosquito monitoring (2011–2016). Parasitol. Res. 116, 3253–3263. 10.1007/s00436-017-5619-3 29032497

[B34] Knight K. L. (1947). The Aedes (Finlaya) chrysolineatus group of mosquitoes (Diptera: Culicidae). Ann. Entomol. Soc. Am. 40, 624–649. 10.1093/aesa/40.4.624

[B35] Knight K. L. (1968). Contributions to the Mosquito Fauna of Southeast Asia IV: Species of the Subgroup Chrysolineatus of Group D, Genus Aedes, Subgenus Finlaya Theobald, Vol. 2. American Entomological Institute. p. 1–45.

[B36] Kovalenko I. Tikhonov S. (2019). *Aedes koreicus* (Edwards, 1917)(Diptera, Culicidae) Recorded in Crimea. Entomol. Rev. 99, 388–392. 10.1134/S0013873819030102

[B37] Kurucz K. Kiss V. Zana B. Jakab F. Kemenesi G. (2018). Filarial nematode (order: Spirurida) surveillance in urban habitats, in the city of Pécs (Hungary). Parasitol. Res. 117, 3355–3360. 10.1007/s00436-018-6066-5 30196322

[B38] Kurucz K. Kiss V. Zana B. Schmieder V. Kepner A. Jakab F.. (2016). Emergence of *Aedes koreicus* (Diptera: Culicidae) in an urban area, Hungary, 2016. Parasitol. Res. 115, 4687–4689. 10.1007/s00436-016-5229-5 27511369

[B39] Lee S. E. Kim H. C. Chong S. T. Klein T. A. Lee W. J. (2007). Molecular survey of *Dirofilaria immitis* and *Dirofilaria repens* by direct PCR for wild caught mosquitoes in the Republic of Korea. Vet. Parasitol. 148, 149–155. 10.1016/j.vetpar.2007.04.015 17644255

[B40] Magnifico I. Petronio Petronio G. Venditti N. Cutuli M. A. Pietrangelo L. Vergalito F.. (2020). Atopic dermatitis as a multifactorial skin disorder. Can the analysis of pathophysiological targets represent the winning therapeutic strategy? Pharmaceuticals 13, 411. 10.3390/ph13110411 33266440 PMC7700401

[B41] Marano G. Pupella S. Pati I. Masiello F. Franchini M. Vaglio S.. (2017). Ten years since the last Chikungunya virus outbreak in Italy: history repeats itself. Blood Transf. 15, 489. 10.2450/2017.0215-17 29053100 PMC5649955

[B42] Marcantonio M. Metz M. Baldacchino F. Arnoldi D. Montarsi F. Capelli G.. (2016). First assessment of potential distribution and dispersal capacity of the emerging invasive mosquito *Aedes koreicus* in Northeast Italy. Parasit. Vect. 9, 1–19. 10.1186/s13071-016-1340-9 26842546 PMC4739402

[B43] Marini G. Arnoldi D. Baldacchino F. Capelli G. Guzzetta G. Merler S.. (2019). First report of the influence of temperature on the bionomics and population dynamics of *Aedes koreicus*, a new invasive alien species in Europe. Parasit. Vect. 12, 1–12. 10.1186/s13071-019-3772-5 31694685 PMC6833271

[B44] McCormack D. R. Salata K. F. Hershey J. N. Carpenter G. B. Engler R. J. (1995). Mosquito bite anaphylaxis: immunotherapy with whole body extracts. Ann. Allerg. Asthma Immunol 74, 39–44. 7719881

[B45] Medlock J. Hansford K. Versteirt V. Cull B. Kampen H. Fontenille D.. (2015). An entomological review of invasive mosquitoes in Europe. Bull. Entomol. Res. 105, 637–663. 10.1017/S0007485315000103 25804287

[B46] Mendoza-Roldan J. A. Gabrielli S. Cascio A. Manoj R. R. Bezerra-Santos M. A. Benelli G.. (2021). Zoonotic Dirofilaria immitis and *Dirofilaria repens* infection in humans and an integrative approach to the diagnosis. Acta Trop. 223, 106083. 10.1016/j.actatropica.2021.106083 34364896

[B47] Miles J. (1964). Some ecological aspects of the problem of arthropod-borne animal viruses in the Western Pacific and South-East Asia regions. Bull. World Health Org. 30, 197. PMC2554795 14153409

[B48] Miyagi I. (1971). Notes on the Aedes (Finlaya) chrysolineatus subgroup in Japan and Korea (Diptera: Culicidae). Trop. Med. 13, 141–151.

[B49] Montarsi F. Ciocchetta S. Devine G. Ravagnan S. Mutinelli F. di Regalbono A. F.. (2015a). Development of *Dirofilaria immitis* within the mosquito *Aedes* (Finlaya*) koreicus*, a new invasive species for Europe. Parasit. Vectors 8, 1–9. 10.1186/s13071-015-0800-y 25884876 PMC4382832

[B50] Montarsi F. Drago A. Dal Pont M. Delai N. Carlin S. Cazzin S.. (2014). Current knowledge on the distribution and biology of the recently introduced invasive mosquito *Aedes koreicus* (Diptera: Culicidae). Firenze 62, 169–174. 10.13140/RG.2.1.2716.5925

[B51] Montarsi F. Drago A. Martini S. Calzolari M. De Filippo F. Bianchi A.. (2015b). Current distribution of the invasive mosquito species, *Aedes koreicus* [Hulecoeteomyia koreica] in northern Italy. Parasit. Vectors 8, 1–5. 10.1186/s13071-015-1208-4 26626019 PMC4666031

[B52] Montarsi F. Martini S. Dal Pont M. Delai N. Milone N. F. Mazzucato M.. (2013). Distribution and habitat characterisation of the recently introduced invasive mosquito *Aedes koreicus* [Hulecoeteomyia koreica], a new potential vector and pest in north-eastern Italy. Parasit. Vectors 6, 1–10. 10.1186/1756-3305-6-292 24457085 PMC3852218

[B53] Negri A. Arnoldi I. Brilli M. Bandi C. Gabrieli P. Epis S. (2021). Evidence for the spread of the alien species *Aedes koreicus* in the Lombardy region, Italy. Parasit. Vectors 14, 1–6. 10.1186/s13071-021-05031-7 34649599 PMC8515701

[B54] Pampiglione S. Rivasi F. Angeli G. Boldorini R. Incensati R. Pastormerlo M.. (2001). Dirofilariasis due to *Dirofilaria repens* in Italy, an emergent zoonosis: report of 60 new cases. Histopathology 38, 344–354. 10.1046/j.1365-2559.2001.01099.x 11318900

[B55] Peng Z. Rasic N. Liu Y. Simons F. E. R. (2002). Mosquito saliva–specific IgE and IgG antibodies in 1059 blood donors. J. Allerg. Clin. Immunol. 110, 816–817. 10.1067/mai.2002.128736 12417897

[B56] Pfitzner W. P. Lehner A. Hoffmann D. Czajka C. Becker N. (2018). First record and morphological characterisation of an established population of *Aedes* (Hulecoeteomyia) *koreicus* (Diptera: Culicidae) in Germany. Parasit. Vectors 11, 1–10. 10.1186/s13071-018-3199-4 30558660 PMC6296035

[B57] Purse B. V. Mellor P. S. Rogers D. J. Samuel A. R. Mertens P. P. Baylis M. (2005). Climate change and the recent emergence of bluetongue in Europe. Nat. Rev. Microbiol. 3, 171–181. 10.1038/nrmicro1090 15685226

[B58] Shestakov V. I. Mikheeva A. I. (1966). Study of vectors of Japanese encephalitis in the Maritime Territory]. Med Parazitol (Mosk) 35, 545–550. 4305799

[B59] Silva L. A. Dermody T. S. (2017). Chikungunya virus: epidemiology, replication, disease mechanisms, and prospective intervention strategies. J. Clin. Investig. 127, 737–749. 10.1172/JCI84417 28248203 PMC5330729

[B60] Simón F. Siles-Lucas M. Morchón R. González-Miguel J. Mellado I. Carretón E.. (2012). Human and animal dirofilariasis: the emergence of a zoonotic mosaic. Clin. Microbiol. Rev. 25, 507–544. 10.1128/CMR.00012-12 22763636 PMC3416488

[B61] Suter T. Flacio E. Fariña B. F. Engeler L. Tonolla M. Müller P. (2015). First report of the invasive mosquito species *Aedes koreicus* in the Swiss-Italian border region. Parasit. Vectors 8, 1–4. 10.1186/s13071-015-1010-3 26223377 PMC4520022

[B62] Tanaka K. Mizusawa K. Saugstad E. S. (1979). A revision of the adult and larval mosquitoes of Japan (including the Ryukyu Archipelago and the Ogasawara Islands) and Korea (Diptera: Culicidae), Vol. 16. Army Medical Lab Pacific Apo San Francisco. p.987.

[B63] Teekema S. Stroo A. Uiterwijk M. van de Vossenberg B. Jacobs F. Ibáñez-Justicia A. (2022). First finding of *Aedes koreicus* (Diptera: Culicidae) in the Netherlands. *J. Eur. Mosq. Cont. Assoc*. 1–8. 10.52004/JEMCA2021.0005

[B64] Tripepi L. (2014). Preferenze alimentari di Aedes koreicus, una nuova zanzara invasiva e implicazioni nella trasmissione di patogeni (Master's thesis). Università degli studi di Padova, Padova, Italy.

[B65] Versteirt V. De Clercq E. M. Fonseca D. Pecor J. Schaffner F. Coosemans M.. (2014). Bionomics of the established exotic mosquito species Aedes koreicus in Belgium, Europe. J. Med. Entomol. 49, 1226–1232. 10.1603/ME11170 23270149

[B66] Versteirt V. Pecor J. E. Fonseca D. M. Coosemans M. Van Bortel W. (2012). Confirmation of *Aedes koreicus* (Diptera: Culicidae) in Belgium and description of morphological differences between Korean and Belgian specimens validated by molecular identification. Zootaxa 3191, 21–32. 10.11646/zootaxa.3191.1.2

[B67] Werner D. Zielke D. E. Kampen H. (2016). First record of *Aedes koreicus* (Diptera: Culicidae) in Germany. Parasitol. Res. 115, 1331–1334. 10.1007/s00436-015-4848-6 26614356

[B68] WHO. (2022). Zika epidemiology update - February 2022. Available online at: https://www.who.int/publications/m/item/zika-epidemiology-update—february-2022 (accessed 2 February 2022).

[B69] Yamada S. (1928). An experimental Study on twenty-four Species of Japanese Mosquitoes regarding their Suitability as intermediate Hosts for *Filaria bancrofti* Cobbold. Sci. Rep. Govt. Inst. Infect, Dis. 6, 559–622.

[B70] Yavuz S. T. Akin O. Koc O. Güngör A. Bolat A. Gülec M. (2021). Mosquito hypersensitivity may be associated with atopic background in children. Allerg. Immunopathol. 49, 67–72. 10.15586/aei.v49i6.448 34761660

[B71] Zeller H. Van Bortel W. Sudre B. (2016). Chikungunya: its history in Africa and Asia and its spread to new regions in 2013–2014. J. Infect. Dis. 214, S436–S440. 10.1093/infdis/jiw391 27920169

[B72] Zotzmann S. Steinbrink A. Cunze S. Klimpel S. (2019). *Aedes koreicus*—a new member of the genus *Aedes* establishing in Germany? Parasitol. Res. 118, 1073–6. 10.1007/s00436-019-06232-x 30734861

