# Supplementary material for: The new invasive mosquito species Aedes koreicus as vector-borne diseases in the European area, a focus on Italian region: What we know from the scientific literature
Source: Front Microbiol. 2022 Jul 25;13:931994. doi: 10.3389/fmicb.2022.931994 (PMC9358684; doi:10.3389/fmicb.2022.931994)
Supplement: Supplementary file 1 [file Data_Sheet_1.PDF]

Supplementary Material

1 Supplementary Figure

Supplementary Figure 1. *Search and selection criteria for scientific articles revision*

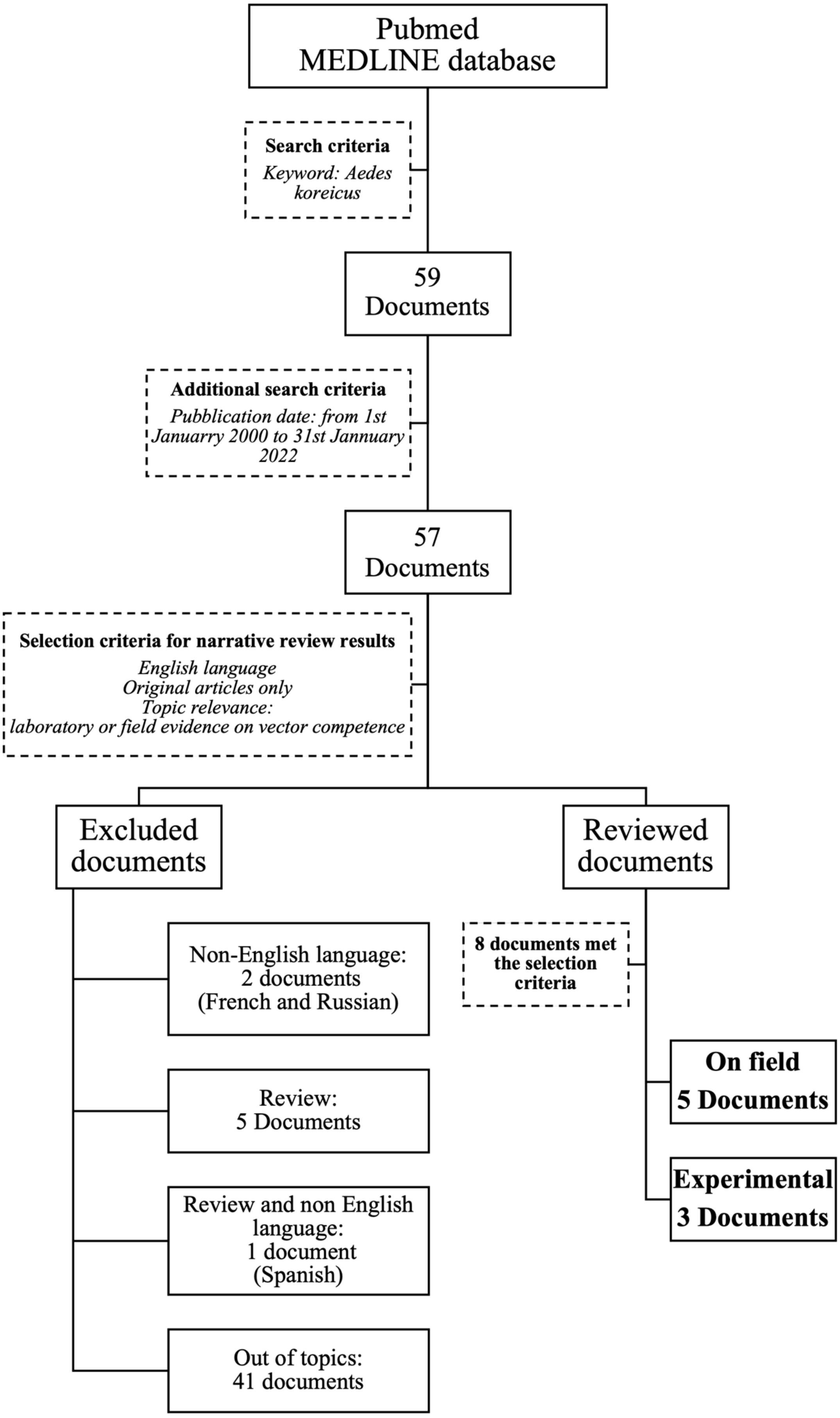
Pubmed
MEDLINE database
Search criteria
Keyword: Aedes koreicus
59 Documents
Additional search criteria
Pubblication date: from 1st Januarry 2000 to 31st Jannuary 2022
57 Documents
Selection criteria for narrative review results
English language
Original articles only
Topic relevance:
laboratory or field evidence on vector competence
Excluded documents
Reviewed documents
Non-English language: 2 documents (French and Russian)
Review: 5 Documents
Review and non English language: 1 document (Spanish)
Out of topics: 41 documents
8 documents met the selection criteria
On field 5 Documents
Experimental 3 Documents

2 Supplementary Table

Supplementary Table 1. *Ae. koreicus* vector carrying capacity, bibliographical findings. MFIR: Minimum Field Infection Rates; JEV: Yellow Fever Virus CHIKV: Chikungunya Virus; ZIKV: Zika virus; WNV: West Nile virus; - not available data. The selected manuscripts were subdivided according to the study type (namely field or experimental) and reviewed according to the publication date (from oldest to most recent).

Collection or experiment execution<br>Year(s)/month(s)	Country/<br>Region(s)	No. mosquitos collected on-field or tested (f= female, t= total)	*Ae. koreicus* no. /pools collected	Evidence type<br>On-field/<br>Experimental	Pathogen(s)<br>Species	 MFIR/<br>positives no.	Method(s) used for pathogen(s) identification	Study aims	Main findings, conclusions, and remarks	Reference
					**Field studies**					
2005/May to September	Republic of Korea/Gyeonggi Province and Gangwon Province	2059 (t)	4/3	Field	*Dirofilaria immitis*	0/0	PCR	Evaluation as potential vectors for *D. immitis* and *D. repens*	Larger studies including seasonal infections are needed for proper vector determination	(Lee et al., 2007)]
					Dirofilaria repens	0/0				
					D. immitis/ repens	0/0				
2009/ May to October	Republic of Korea/ Geomun-ri, Geomun lsland, Yeosu-si and Maejuk-ri, Maemul lsland, Tongyoung-si	5380 (t)	3/-	Field	*Brugia malayi.*	-/0	PCR	Surveillance of mosquitoes as vectors of filariasis	The survey did not detect filarial DNA in mosquitoes from Korean endemic areas	(Cho et al., 2012)
2016-2017	Hungary/ Baranya County, Pécs urban area	1123 (t)	68/46	Field	*D. repens*	-/25	PCR	To assess the prevalence and distribution	More attention should be paid to the zoonotic potential of	(Kurucz et al., 2018)

								of filariae in field-collected mosquitoes	*Dirofilaria a*lso for human clinicians.	
2015-2018	Italy/Liguria	4499 (t)	62/26	Field	*Flavivirus*	-/0	RT-PCR	Reports the finding of *Ae. koreicus* in Genoa over four consecutive years	*Ae. koreicus* has become established in critical areas. All *Ae. koreicus* analysed were negative for flaviviruses.	(Ballardini et al., 2019)
2016–2018/March to November	Republic of Korea/Incheon Metropolitan City and Hwaseong-Gun Area	35445 (f)	594/56	Field	WNV, JEV, and dengue fever virus.	0	RT-qPCR	Epidemiological and risk assessment evidence of mosquito-borne pathogens	Climate variability can have a direct influence on vector-borne diseases epidemiology	(Jegal et al., 2020)
**Experimental studies**										
2014	Italy/Veneto	-	54/-	Experimental	*D. immitis*	32	Artificial feeding system	Assessment of filaria *larvae* development	Experimental results demonstrated the involvement of *Ae. koreicus* in the life cycle of *D. immitis*	(Montarsi et al., 2015a)
2018	Italy/Veneto	-	342/-	Experimental	CHIKV strain La Reunion	-	*Ex vivo* infection under constant and fluctuating temperature regimes	To provide preliminary data on the potential of *Ae. koreicus* to transmit CHIKV	Low-level virus transmission is possible in regions with temperatures similar to those tested.	(Ciocchetta et al., 2018)
2018-2019	Germany/ Hamburg	-	20/-	Experimental	CHIKV, ZIKV and WNV	-	Experimental infections and RT-qPCR	To estimate the competence of field-caught Ae. koreicus as vectors for different arboviruses	CHIKV and ZIKV, but no WNV potential trasmission. A new insect-specific	(Jansen et al., 2021)

						virus was identified.	
